# Protective Role for LPA_3_ in Cardiac Hypertrophy Induced by Myocardial Infarction but Not by Isoproterenol

**DOI:** 10.3389/fphys.2017.00356

**Published:** 2017-05-30

**Authors:** Lin Cai, Guangpu Fan, Fang Wang, Si Liu, Tiewei Li, Xiangfeng Cong, Jerold Chun, Xi Chen

**Affiliations:** ^1^State Key Laboratory of Cardiovascular Disease, Fuwai Hospital, National Center for Cardiovascular Diseases, Chinese Academy of Medical Sciences and Peking Union Medical CollegeBeijing, China; ^2^Cardiovascular Surgery Department, FuWai Hospital, National Center for Cardiovascular Diseases, Chinese Academy of Medical Sciences and Peking Union Medical CollegeBeijing, China; ^3^Sanford Burnham Prebys Medical Discovery InstituteLa Jolla, CA, United States

**Keywords:** lysophosphatidic acid, LPA_3_, hypertrophy, isoproterenol, MI

## Abstract

**Background:** We previously reported that lysophosphatidic acid (LPA) promoted cardiomyocyte hypertrophy *in vitro* via one of its G protein-coupled receptor subtypes, LPA_3_. In this study, we examined the role of LPA_3_ in cardiac hypertrophy induced by isoproterenol (ISO) and myocardial infarction.

**Methods:** *In vitro*, neonatal rat cardiomyocytes (NRCMs) were subjected to LPA_3_ knocked-down, or pretreated with a β-adrenergic receptor (β-AR) antagonist (propranolol) before LPA/ISO treatment. Cardiomyocyte size and hypertrophic gene (ANP, BNP) mRNA levels were determined. *In vivo*, ${\text{LPA}}_{3}^{-/-}$ and wild-type mice were implanted subcutaneously with an osmotic mini-pump containing ISO or vehicle for 2 weeks; echocardiography was performed to determine the heart weight/body weight ratio, cardiomyocyte cross-sectional area, and level of ANP mRNA expression. ${\text{LPA}}_{3}^{-/-}$ and wild-type mice were subjected to permanent coronary artery ligation or sham surgery for 4 weeks; cardiac function, including the degree of hypertrophy and infarction size, was determined.

**Results:** *In vitro*, we found that knocked-down LPA_3_ in NRCMs did not attenuate ISO-induced hypertrophy, and propranolol was unable to abolish LPA-induced hypertrophy. *In vivo*, chronic ISO infusion caused cardiac hypertrophy in wild-type mice, while hypertrophic responses to ISO infusion were not attenuated in ${\text{LPA}}_{3}^{-/-}$ mice. However, in a myocardial infarction (MI) model, ${\text{LPA}}_{3}^{-/-}$ mice exhibited reduced cardiac hypertrophy compared to wild-type mice at 4 weeks post-MI, which was associated with reduced cardiac function and increased infarct size.

**Conclusions:** Our data show that LPA_3_ appears to play a protective role in myocardial hypertrophy post-MI, but does not appear to be involved in the hypertrophy that occurs in response to β-AR stimulation *in vivo* and *in vitro*. These results implicate LPA-LPA_3_ lipid signaling in cardiac hypertrophy occurring after pathological insults like MI, which presents a new variable in β-AR-independent hypertrophy. Thus, modulation of LPA_3_ signaling might represent a new strategy for preventing the stressed myocardium from ischemia injury.

## Introduction

Pathological cardiac hypertrophy is a powerful and independent risk factor for heart failure and mortality (Haider et al., 1998; Okin et al., 2006). β-adrenergic receptor (β-AR) stimulation and the renin-angiotensin-aldosterone system (RAAS) are both thought to be involved in this condition (Gupta et al., 2007). However, although β-AR blockade and angiotensin-converting enzyme inhibition are associated with an improvement in left ventricular performance and the reversal of left ventricular remodeling, left ventricular systolic function can still deteriorate, resulting in a high incidence of heart failure that is a huge burden on healthcare resources (Ma et al., 2010; Dinicolantonio et al., 2015; Mozaffarian et al., 2016). New mechanisms need to be identified in order to better understand and treat both cardiac hypertrophy and the associated heart failure.

Lysophosphatidic acid (LPA) is a small glycerophospholipid, which is involved in multiple biological actions including proliferation, survival, migration, apoptosis, and differentiation via its six, seven-transmemberane G protein-coupled receptors (GPCRs), LPA_1_–LPA_6_ (Choi et al., 2010; Kihara et al., 2014; Yung et al., 2014). Our previous study found that cardiomyocyte hypertrophy could be induced by LPA, via Akt and ERK-NFκB signaling pathways (Chen et al., 2008). Moreover, we have recently identified pro-hypertrophic effects of LPA on neonatal rat cardiomyocytes (NRCMs) are mediated through the LPA_3_ receptor (encoded by *Lpar3*) (Yang et al., 2013b). However, whether hypertrophic signaling induced by LPA-LPA_3_ is the same or different from other well-established pro-hypertrophic pathways, and whether the role of LPA_3_ in ventricular remodeling is good or bad, are currently unknown. In the present study, we investigated whether LPA-LPA_3_ is involved in hypertrophy induced by β-AR activation *in vitro* and *in vivo*, and assessed possible roles for LPA_3_ signaling in the heart following myocardial infarction (MI) in mice.

## Materials and methods

### Mice

The ${\text{LPA}}_{3}^{-/-}$ mice used in this study were described previously (Yang et al., 2002; Ye et al., 2005; Choi et al., 2008). All mice were bred from a BALBc strain, and backcrossed for more than 10 generations. This study was carried out in accordance with the “Regulation to the Care and Use of Experimental Animals” of the Beijing Council on Animal Care study (1996). The protocol was approved by the Fuwai Hospital Animal Care and Use Committee.

### Primary culture of neonatal rat cardiomyocytes

The culture of NRCMs isolated from 1- to 3-day-old Sprague-Dawley rats was carried out as previously described (Chen et al., 2002). DMEM containing 10% fetal bovine serum, penicillin/streptomycin (1,000 U/ml each) with 100 mM 5-Bromo-2-deoxyUridine was added to inhibit the growth of cardiac fibroblasts. After 24 h of culture, the cells were washed and starved overnight in serum-free medium prior to use within experiments.

### Transfection of siRNA for LPA_3_

Small interfering RNA (siRNA) (Invitrogen, Carlsbad, CA, USA) for LPA_3_ was transfected into cardiomyocytes using Lipofectamine™ RNAiMAX according to the manufacturer's protocol, as previously described (Yang et al., 2013b). Stealth siRNA duplex target sequences were 5′-UACACCACCACCAUGAUGAAGAAGG-3′ and 5′-CCUUCUUCAUCAUGGUGGUGGUGUA-3′. The sequence for the stealth siRNA low-GC duplex was used as a negative control. The cells were transfected with the stealth siRNA at 20 nM.

### Immunofluorescence staining

NRCMs were placed on coverslips in six-well culture plates and treated with LPA (5 μM) or ISO (1 μM) for 24 h, or pretreated with propranolol (1 μM) for half an hour before LPA/ISO stimulation. In the transfection experiments, cells were transfected with siRNA of LPA_3_ or the negative control for 24 h, then exposed to LPA or ISO for 24 h. The proportion of cells staining positively for α-actinin was measured to determine the degree of cardiomyocyte hypertrophy. For α-actinin staining, cells were fixed with 4% paraformaldehyde for 30 min, and subsequently permeabilized with 0.1% Triton X-100 for 10 min and 1% BSA for 30 min at room temperature. After washing, cells were incubated with mouse monoclonal antibody against α-actinin (1:250, Abcam, Cambridge, MA, USA) at 37°C for 2 h and a fluorescein-conjugated goat-anti-mouse IgG (1:300, Zhongshan Jinqiao Biotechnology, Beijing, China) at 37°C for 1 h. DAPI staining marked the nuclei. The cells were visualized under a fluorescence microscope (Zeiss, Oberkochen, Germany) and laser confocal microscopy (Leica, Wetzlar, Germany). At least 100 cardiomyocytes in 20–30 fields were examined in each group.

### Mouse model of ISO-induced hypertrophy

Micro-osmotic pumps (model 1002, Alzet) releasing ISO (Sigma, St. Louis, MO, USA; 60 mg/kg/day in PBS) or vehicle (PBS) were implanted subcutaneously into age-matched (8–10 weeks old) male ${\text{LPA}}_{3}^{-/-}$ mice and wild-type littermates after being anesthetized by intraperitoneal administration of tribromoethanol (400 mg/kg). Hypertrophy was assessed by echocardiography using a VisualSonics Vevo 2100 ultrasound system (VisualSonics, Inc., Toronto, ON, Canada). Ventricular measurements in M-mode were taken before and 13 days after implantation. The following day mice were sacrificed for histological and molecular analysis.

### Myocardial infarction

Myocardial infarction (MI) experiments were performed on 8–10 week-old ${\text{LPA}}_{3}^{-/-}$ and wild-type mice. The MI model was characterized as previously described (Fan et al., 2015). Mice were anesthetized by intraperitoneal administration of tribromoethanol (400 mg/kg) and ventilated with a rodent respirator, then the left anterior descending coronary artery was permanently occluded using a 7-0 polypropylene suture, and the occlusion was confirmed by blanching of the anterior wall of the left ventricle. For non-infarcted controls, mice underwent sham operation where the ligature around the left anterior descending coronary artery was not tied. Animals were recovered from anesthesia under warm conditions with normal ventilation. Four weeks after surgery, cardiac function was assessed by echocardiography using a VisualSonics Vevo 2100 ultrasound system (VisualSonics, Inc.), then animals were sacrificed and hearts were excised for further analysis.

### Morphological examination and measurement of infarct size

Hearts were fixed in 10% formalin overnight at room temperature and embedded in paraffin. Subsequently, the hearts were cut serially from the apex to the base. Each short-axis section (5 μm), collected with an interval of 200 μm, was stained with Picrosirius red for morphometric analysis. The experiments were executed following standard procedures. The infarct size was calculated as a percentage of the total left ventricle (LV) wall circumference from each of the three LV sections. Scar circumstance was calculated using Image Analysis Software.

### Wheat germ agglutinin (WGA) staining

WGA staining was carried out to measure the cross-sectional area of cardiac myocytes. Following antigen retrieval using citric acid buffer (10 mM, pH 6.0), the sections were blocked with 1% bovine serum albumin for 30 min at room temperature and then incubated with Alexa Fluor®594-WGA (Invitrogen) for 1 h at room temperature. The slides were washed three times in PBS, mounted using an anti-fade mounting media containing DAPI (Zhongshan Jinqiao Biotechnology), and imaged was performed using a Leica DM6000B microscope.

### Quantitative real-time PCR

Total RNA was extracted from mice ventricular tissue and cultured cardiomyocytes using Trizol, and was then quantified using a Nanodrop 2000 spectrophotometer. cDNA was generated from total RNA (1 μg) using M-MLV reverse transcriptase and oligo(dT)15 primer. qRT-PCR was performed using a SYBR Green PCR Master Mix and an Applied Biosystems 7500 (ABI, Foster City, CA, USA). The primer pairs used in this study are listed in Table 1.

**Table 1:** Primers for quantitative real-time PCR detection.

**Gene**	**Sequence 5′— 3′**	**Species**
LPA_3_	F: TGTCAACCGCTGGCTTCT	Rat
	R: CAGTCATCACCGTCTCATTAG	
ANP	F: AGGAGAAGATGCCGGTAGAAGA	Rat
	R: GCTTCCTCAGTCTGCTCACTCA	
BNP	F: TAGCCAGTCTCCAGAGCAATTC	Rat
	R: TTGGTCCTTCAAGAGCTGTCTC	
GAPDH	F: AAATGGTGAAGGTCGGTGTGAAC	Rat
	R: CAACAATCTCCACTTTGCCACTG	
ANP	F: AGGAGAAGATGCCGGTAGAAGA	Mouse
	R: GCTTCCTCAGTCTGCTCACTCA	
GAPDH	F: CAACGACCCCTTCATTGACCT	Mouse
	R: CAGTAGACTCCACGACATACTC	

### Statistical analysis

All data are presented as mean ± SEM. Differences among groups were assessed by a one-way analysis of variance (ANOVA) followed by a *post-hoc* Tukey's test. Comparisons between two groups were performed using a Student's *t*-test. All statistical analyses were performed with GraphPad Prism 6.0. A value of *p* < 0.05 was considered to indicate a statistically significant difference.

## Results

### LPA_3_ interference does not reduce ISO-induced cardiomyocyte hypertrophy

**Figure 1:**
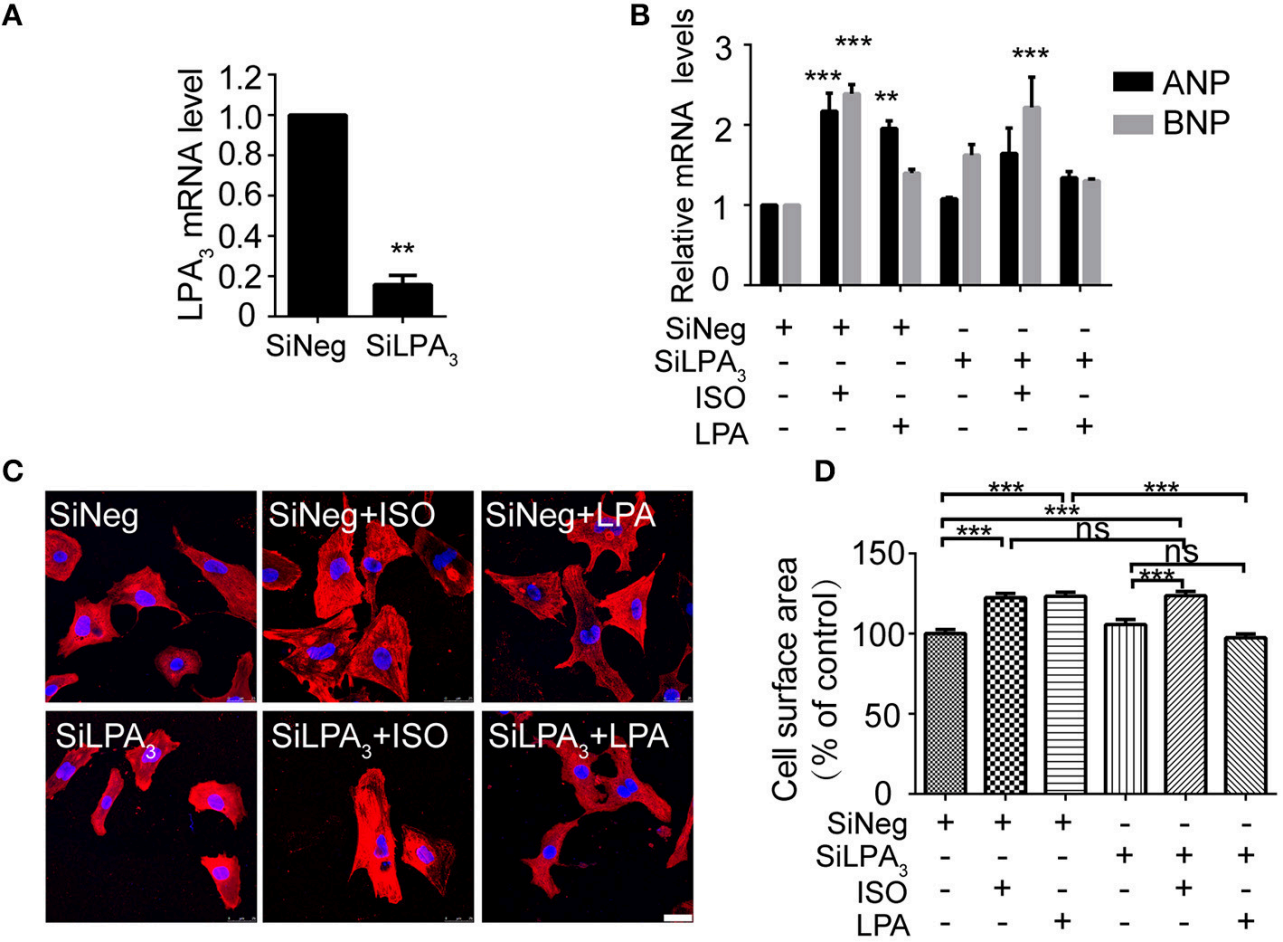
LPA_3_ silencing does not abolish ISO-induced cardiomyocyte hypertrophy. Rat neonatal cardiomyocytes were transfected with siRNA of LPA_3_ for 24 h. The level of LPA_3_ mRNA was measured by qPCR **(A)**. After knockdown of LPA_3_, NRCMs were treated with ISO or LPA for 24h. The mRNA levels for pro-hypertrophic genes (ANP and BNP) were compared in the indicated groups **(B)**. NRCMs were stained using an α-actinin antibody and DAPI to measure the average cell surface area (**C,D**, scale bar, 100 μm. Over 100 individual cells were analyzed per group). ^**^p < 0.01, ^***^p < 0.001, ns *p* > 0.05 vs. SiNeg.

To test whether LPA_3_ is involved in an agonist of β-AR, ISO-induced hypertrophy, we used RNAi technology to knock down LPA_3_ expression in neonatal rat cardiomyocytes (Figure 1A). Cardiomyocyte size and the expression of atrial natriuretic factor (ANP) and brain natriuretic factor (BNP) mRNA were significantly increased after LPA or ISO treatment (Figures 1B–D). LPA_3_ interference resulted in a significant reduction in LPA-induced cardiomyocyte hypertrophy, but had no effect on ISO-induced ANP and BNP mRNA expression (Figure 1B) or cardiomyocyte size (Figures 1C,D). These results suggest that LPA_3_ is not involved in ISO-induced cardiomyocyte hypertrophy *in vitro*.

### β-AR antagonist does not abolish LPA-induced cardiomyocyte hypertrophy

**Figure 2:**
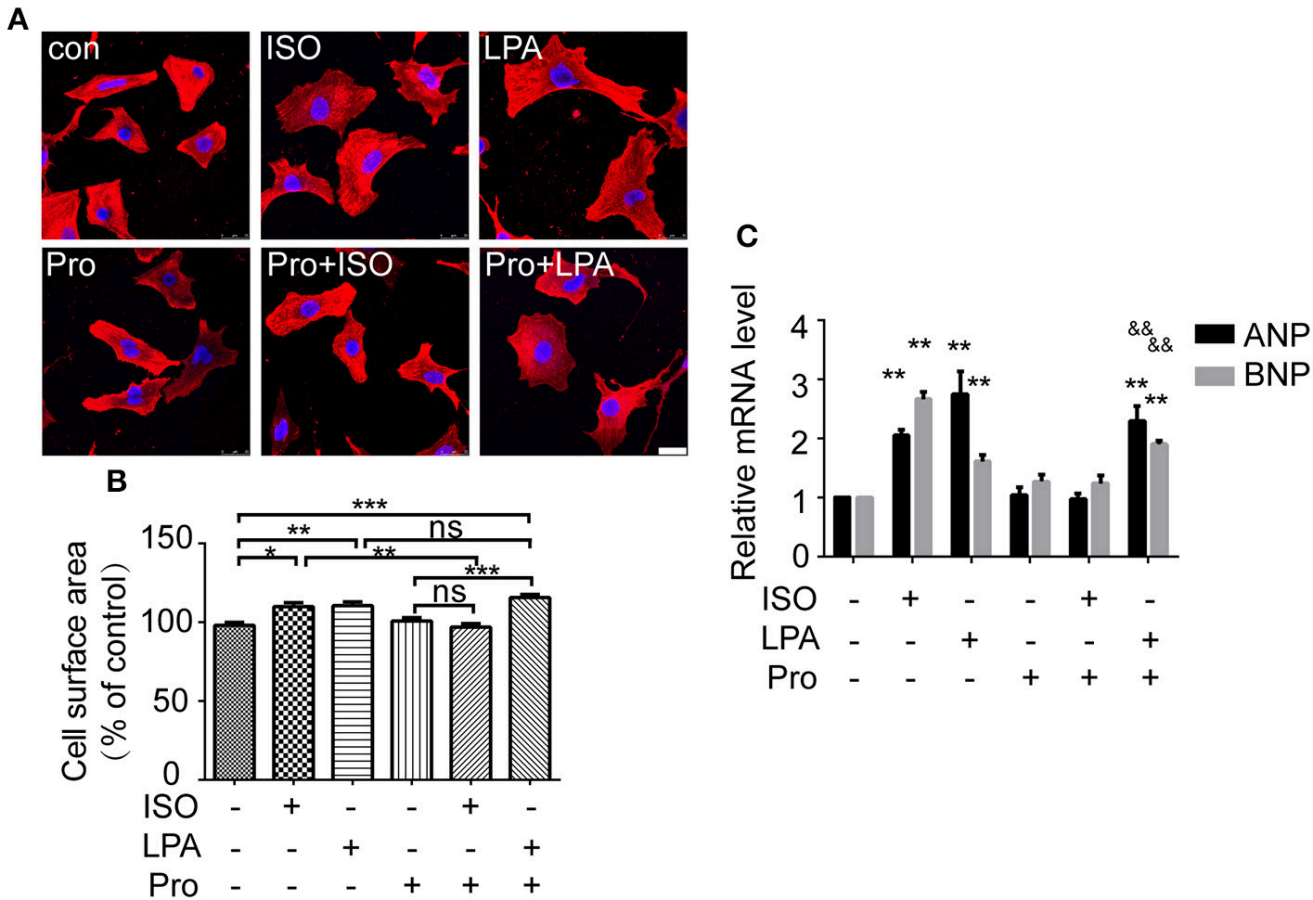
LPA-induced cardiomyocyte hypertrophy is not affected by a β-adrenergic receptor antagonist. Serum-starved NRCMs were treated with propranolol (Pro) for 0.5 h before LPA or ISO stimulation for 24 h. NRCMs were stained with an α-actinin antibody and DAPI to measure the average cell surface area (**A,B**, scale bar, 100 μm, over 100 individual cells were analyzed per group). The mRNA levels for pro-hypertrophic genes (ANP and BNP) were compared in the indicated groups **(C)**. ^*^p < 0.05, ^**^p < 0.01, ^***^p < 0.001, ns *p* > 0.05 vs. control. &&*p* < 0.01 vs. Pro.

To further explore whether the β-AR participates in LPA-induced cardiomyocyte hypertrophy, a β-AR antagonist, propranolol, was used. After propranolol (1 μM) pretreatment for 30 min, ISO failed to increase cardiomyocyte size and ANP and BNP mRNA levels. However, LPA-induced cardiomyocyte hypertrophy was not significantly inhibited (Figures 2A–C). Taken together, our results indicate that ISO and LPA promote cardiomyocyte hypertrophy *in vitro* by independent mechanisms.

### LPA_3_-deficiency does not affect ISO-induced cardiac hypertrophy *in vivo*

**Figure 3:**
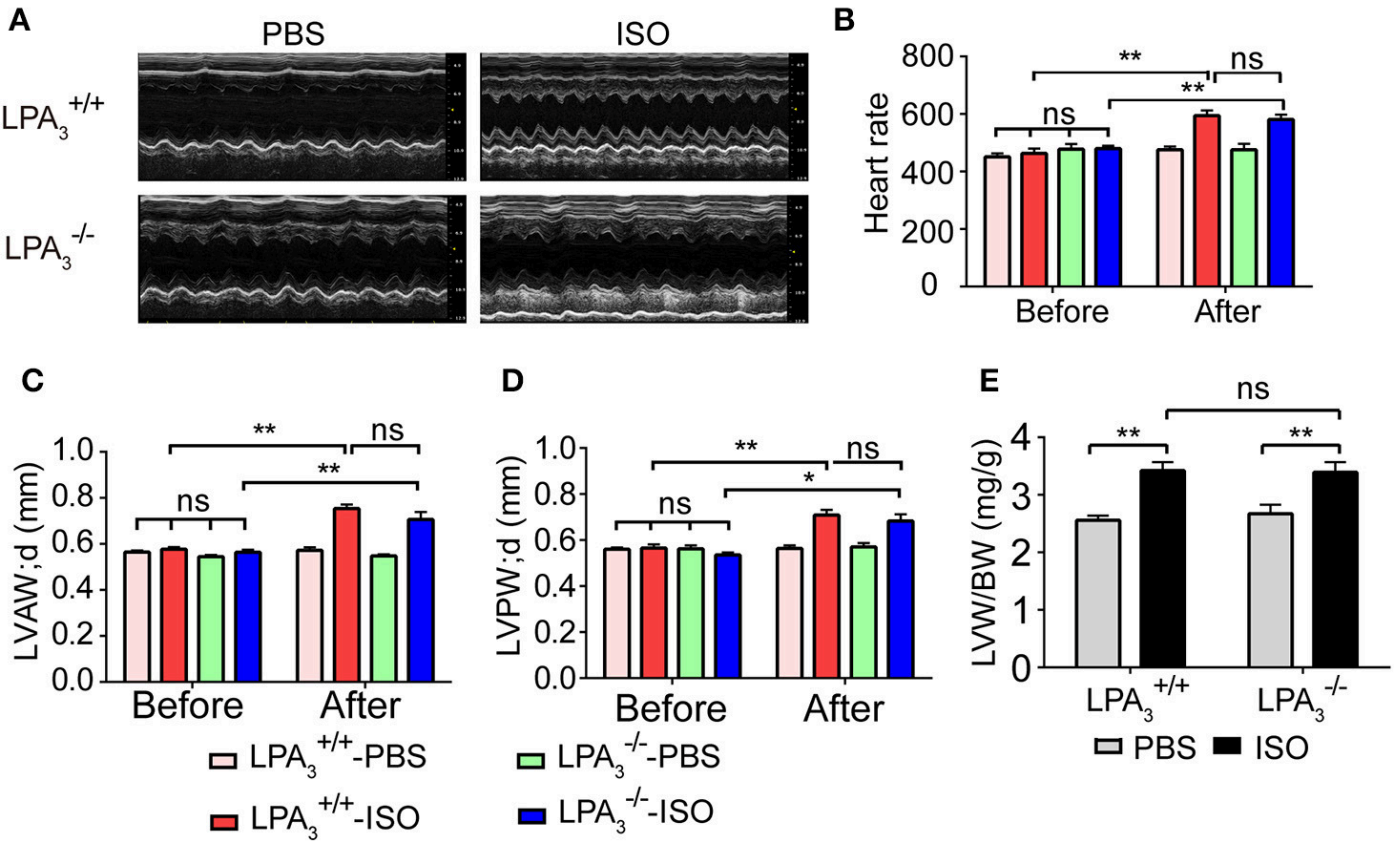
Loss of LPA_3_ does not affect echocardiographic parameters associated with ISO-induced cardiac hypertrophy. After ISO (60 mg/kg/d in PBS) or vehicle (PBS) infusion for 2 weeks, M-mode echocardiograms of hearts from wild-type or LPA_3_ knockout mice were recorded. Representative examples are shown in **(A)**. Echocardiographic parameters including heartrate **(B)**, LV diastolic anterior wall (LVAWd) **(C)**, LV diastolic posterior wall (LVPWd) **(D)**, and the left ventricular weight/body weight ratio **(E)** were measured. *N* = 8–11 mice per group. ^*^p < 0.05, ^**^p < 0.01, ns *p* > 0.05.

We further assessed whether the absence of LPA_3_ attenuates ISO-induced cardiac hypertrophy *in vivo*. ${\text{LPA}}_{3}^{-/-}$ and wild-type mice were subcutaneously implanted with an osmotic pump delivering ISO (60 mg/kg/day) for 2 weeks. Echocardiology data showed that left ventricular anterior and posterior wall thickness, and the left ventricular weight/body weight ratio were significantly increased in wild-type mice after ISO infusion (Figures 3A,C–E). ${\text{LPA}}_{3}^{-/-}$ mice showed similar responses to ISO infusion. Furthermore, heart rate was significantly increased in both wild-type and ${\text{LPA}}_{3}^{-/-}$ mice after ISO infusion (Table 2, Figure 3B).

**Table 2:** Echocardiographic characteristics of the different study groups at baseline and 2 weeks post-ISO infusion.

	${\text{LPA}}_{3}^{+/+}$ **Vehicle**		${\text{LPA}}_{3}^{+/+}$ **ISO**		${\text{LPA}}_{3}^{-/-}$ **Vehicle**		${\text{LPA}}_{3}^{-/-}$ **ISO**	
	**Baseline**	**2 weeks**	**Baseline**	**2 weeks**	**Baseline**	**2 weeks**	**Baseline**	**2 weeks**
*N*	11	11	11	11	8	8	8	8
HR	451 ± 12	476 ± 12	463 ± 17	594 ± 18^**^^#^	478 ± 18	476 ± 20	479 ± 11	581 ± 17^**^^#^
BW (g)	22.636 ± 0.329	22.918 ± 0.350	22.718 ± 0.252	23.107 ± 0.431	23.200 ± 0.464	22.833 ± 0.508	23.075 ± 0.233	22.866 ± 0.277
LVAWd (mm)	0.564 ± 0.007	0.572 ± 0.013	0.577 ± 0.009	0.754 ± 0.018^**^	0.545 ± 0.007	0.548 ± 0.007	0.564 ± 0.011	0.707 ± 0.032^*^
LVPWd (mm)	0.561 ± 0.007	0.564 ± 0.013	0.566 ± 0.016	0.709 ± 0.022^**^	0.562 ± 0.015	0.57 ± 0.016	0.535 ± 0.012	0.683 ± 0.030^*^
LVIDd (mm)	3.823 ± 0.083	3.935 ± 0.058	3.927 ± 0.080	3.862 ± 0.094	3.759 ± 0.108	4.053 ± 0.123	3.800 ± 0.066	3.999 ± 0.152
LVIDs (mm)	2.812 ± 0.078	2.852 ± 0.084	2.856 ± 0.069	2.623 ± 0.118	2.694 ± 0.149	3.017 ± 0.135	2.781 ± 0.069	2.772 ± 0.188
EDV (μl)	63.320 ± 3.104	67.570 ± 2.361	67.407 ± 3.208	64.971 ± 3.656	60.893 ± 4.010	72.715 ± 5.366	62.150 ± 2.574	71.021 ± 6.329
ESV (μl)	30.255 ± 1.934	31.614 ± 2.203	31.316 ± 1.754	26.006 ± 2.854	27.831 ± 3.450	36.068 ± 4.123	29.264 ± 1.746	30.381 ± 4.625
EF (%)	52.515 ± 1.362	53.582 ± 2.091	53.575 ± 1.465	60.941 ± 2.444	55.324 ± 3.787	50.926 ± 2.405	53.034 ± 1.617	58.668 ± 3.880
FS (%)	26.523 ± 0.844	27.384 ± 1.403	27.274 ± 0.940	32.353 ± 1.668	28.636 ± 2.518	25.686 ± 1.454	26.846 ± 1.048	31.119 ± 2.786

HR, heart rate; BW, body weight; LVAWd, end-diastolic left ventricular anterior wall thickness; LVPWd, end-diastolic left ventricular posterior wall thickness; LVIDd, end-diastolic left ventricular internal dimension; LVIDs, end-systolic left ventricular internal dimension; EDV, end-diastolic volume; ESV, end-systolic volume; EF, ejection fraction; FS, fractional shortening. One-way analysis of variance (ANOVA) with Tukey's post-hoc test were performed for all groups at each time point.

* *p < 0.05*,

** p < 0.01, 2 weeks vs. baseline within group.

# *p < 0.05 vs. vehicle within group*.

**Figure 4:**
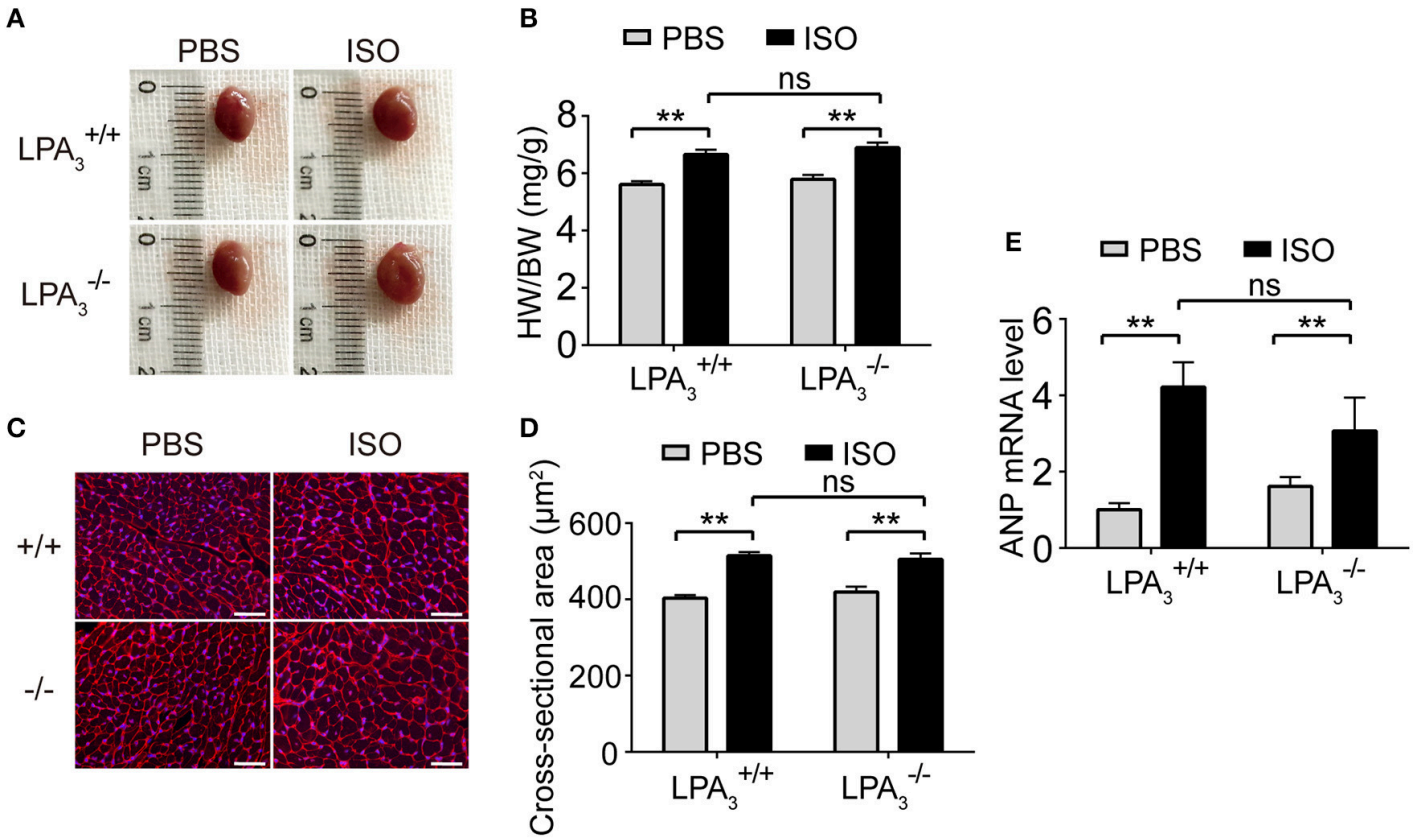
Loss of LPA_3_ does not affect ISO-induced cardiac hypertrophy. After ISO (60 mg/kg/day in PBS) infusion for 2 weeks in wild-type and LPA_3_ knockout mice, heart size **(A)** and the heart weight/body weight ratio (**B**, *n* = 8–11 mice per group) were measured. The cross-sectional area of cardiomyocytes was determined by wheat germ agglutinin staining (**C,D**, scale bar, 50 μm, *n* = 5 mice per group). The level of ANP mRNA expression was compared between the indicated groups (**E**, *n* = 6–8 mice). ^**^p < 0.01, ns *p* > 0.05.

Morphological data show that ISO infusion resulted in a significant increase in heart size (Figure 4A), heart to body ratio (Figure 4B), and cardiomyocyte cross-sectional area in wild-type mice (Figures 4C,D). However, there was no attenuation of these hypertrophic responses in ${\text{LPA}}_{3}^{-/-}$ mice (Figures 4A–D). Furthermore, we assessed the mRNA levels of genes associated with hypertrophy in the four treatment groups. We observed a similar alteration to ANP expression in both ${\text{LPA}}_{3}^{+/+}$ and ${\text{LPA}}_{3}^{-/-}$ hearts after ISO treatment (Figure 4E). These findings support the hypothesis that LPA_3_ deficiency does not affect ISO-induced cardiac hypertrophy *in vivo*.

### LPA_3_-deficiency attenuates cardiac hypertrophy, but aggravates cardiac dysfunction after myocardial infarction

**Figure 5:**
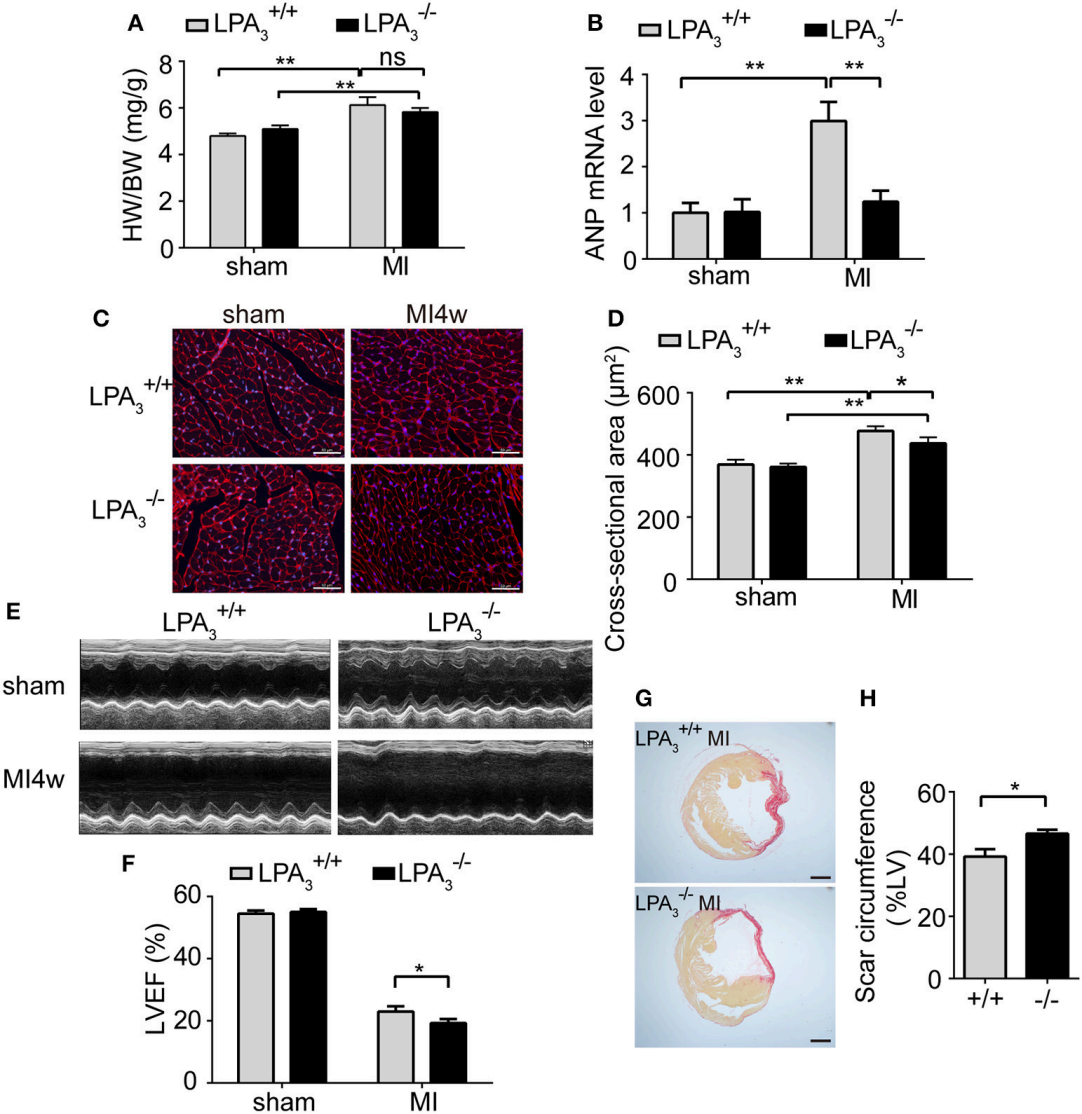
Loss of LPA_3_ attenuates cardiac hypertrophy, but exacerbates infarct size and loss of heart function following an MI. Four weeks after Ml, the heart weight/body weight ratio (**A**, *n* = 13–14 mice per group) and ANP mRNA levels (**B**, *n* = 4 mice per group) were compared in ${\text{LPA}}_{3}^{-/-}$ and wild-type mice. Cardiomyocyte cross-sectional area was determined by wheat germ agglutinin staining. (**C,D**, scale bar, 50 μm, *n* = 5 mice per group). **(E)** Representative echocardiography images and LVEF measurement (**F**, *n* = 13–14 mice per group) in the indicated groups. Scar circumference was measured and expressed as a percentage of the total area of the LV myocardium. Representative images **(G)** and measurement of the scar circumference (**H**, scale bar, 1,000 μm, *n* = 5 mice per group) were determined by Sirius red staining. ^*^p < 0.05, ^**^p < 0.01, ns *p* > 0.05.

Since it is known that LPA-induced hypertrophy is different from the pathological hypertrophy induced by the β-AR, we decided to investigate further the role of LPA_3_-induced hypertrophy in pathological ventricular remodeling. At 4 weeks after MI, ${\text{LPA}}_{3}^{+/+}$ animals showed marked increases in the heart weight/body weight ratio, levels of ANP mRNA expression, and cardiomyocyte cross-sectional area (Figures 5A–D). Compared with ${\text{LPA}}_{3}^{+/+}$ infarcted mice, there was a tendency for a reduced heart weight/body weight ratio (Figure 5A), a notable decrease in ANP mRNA levels (Figure 5B), and a significant decrease in cardiomyocyte cross-sectional area (Figures 5C,D) in ${\text{LPA}}_{3}^{-/-}$ mice post-MI. These results indicate that LPA_3_ is involved in cardiac hypertrophy after MI.

Meanwhile, we further investigated the effect of LPA_3_ deficiency on cardiac function post-MI. ${\text{LPA}}_{3}^{-/-}$ mice manifested no pathological abnormalities with respect to cardiac function at baseline. After MI challenge, the ejection fraction percentage (EF%) was lower in both ${\text{LPA}}_{3}^{+/+}$ and ${\text{LPA}}_{3}^{-/-}$ mice, but ${\text{LPA}}_{3}^{-/-}$ mice exhibited more pronounced LV dilatation and more severe contractile dysfunction (Table 3, Figures 5E,F). Sirius red staining at 4 weeks after MI further displayed a significantly enlarged infarct size in ${\text{LPA}}_{3}^{-/-}$ mice compared with wild-type control animals (*P* < 0.05; Figures 5G,H). These findings suggest that LPA_3_ might play a protective role against MI.

**Table 3:** Echocardiographic characteristics of the different study groups 4 weeks after a myocardial infarction.

	**${\text{LPA}}_{3}^{+/+}$ sham**	**${\text{LPA}}_{3}^{-/-}$ sham**	**${\text{LPA}}_{3}^{+/+}$ MI**	**${\text{LPA}}_{3}^{-/-}$ MI**
*N*	14	14	13	14
HR	445 ± 11	461 ± 10	444 ± 15	450 ± 15
LVAWd (mm)	0.556 ± 0.020	0.542 ± 0.012	0.175 ± 0.017^***^	0.167 ± 0.027^***^
LVPWd(mm)	0.545 ± 0.013	0.548 ± 0.029	0.580 ± 0.033	0.573 ± 0.024
LVIDd (mm)	3.759 ± 0.069	3.831 ± 0.083	4.905 ± 0.115^***^	5.161 ± 0.105^***^^&^
LVIDs (mm)	2.719 ± 0.061	2.800 ± 0.080	4.389 ± 0.126^***^	4.710 ± 0.111^***^^&^
EDV (μl)	60.770 ± 2.522	63.737 ± 3.315	114.169 ± 6.347^***^	128.263 ± 5.809^***^^&^
ESV (μl)	27.226 ± 1.444	30.083 ± 2.105	88.464 ± 6.306^***^	103.975 ± 5.488^***^
EF (%)	54.494 ± 0.993	53.326 ± 1.151	22.993 ± 1.298^***^	19.254 ± 1.298^***^^&^
FS (%)	27.726 ± 0.624	27.031 ± 0.703	10.606 ± 0.811^***^	8.805 ± 1.254^***^

N, number; HR, heart rate; LVAWd, end-diastolic left ventricular anterior wall thickness; LVPWd, end-diastolic left ventricular posterior wall thickness; LVIDd, end-diastolic left ventricular internal dimension; LVIDs, end-systolic left ventricular internal dimension; EDV, end-diastolic volume; ESV, end-systolic volume; EF, ejection fraction; FS, fractional shortening. A one-way analysis of variance (ANOVA) with Tukey's post-hoc test was performed.

*** *p < 0.001 vs. corresponding sham groups*.

& *p < 0.05 vs. LPA_3_^+/+^ MI*.

## Discussion

In the present study, we first demonstrated that LPA_3_ signaling is not involved in ISO-induced cardiac hypertrophy *in vitro* and *in vivo*. However, LPA_3_ deficiency attenuated cardiac hypertrophy while aggravating cardiac dysfunction after MI, suggesting a protective role of LPA_3_ in cardiac function. These results implicate LPA-LPA_3_ lipid signaling as a new variable in cardiac hypertrophy, independent of the β-AR system, which can also affect cardiac function.

Our previous study showed that LPA stimulates cardiomyocyte hypertrophy through LPA_3_ (Yang et al., 2013b). Here we find that hypertrophy induced by LPA-LPA_3_ signaling is independent of ISO-β-AR system. ISO, a catecholamine, is a synthetic β- adrenergic agonist that induces cardiomyocyte hypertrophy in experimental animals (Oudit et al., 2003; Hohimer et al., 2005; Cha et al., 2009). β-AR is one of the most important GPCRs and plays a central role in sympathetic regulation of cardiac function. With sustained ISO stimulation, β-AR binds to Gs protein to elevate cystolic cAMP. cAMP activates PKA to cause abnormal diastolic sarcoplasmic reticulum (SR) Ca^2+^ leak, or directly activate Epac thus inducing NFAT dependent fetal phenotype gene elevation by a CaMKIIδ-dependent manner, which eventually both give rise to hypertrophy and heart failure (Tada and Kirchberger, 1975; Antos et al., 2001; Morel et al., 2005; Metrich et al., 2008; Grimm and Brown, 2010; Ruiz-Hurtado et al., 2012; Pereira et al., 2013). As for LPA-LPA_3_ lipid signaling system, LPA_3_ is also one member of GPCRs. LPA induces cardiomyocyte hypertrophy through a mechanism involving both Gi and the small G protein Rho (Hilal-Dandan et al., 2004), which is different from G proteins that ISO-β-AR couples to. After binding to Gi, LPA_3_ activates Akt and ERK-NFκB, and then promotes the expression of fetal phenotype gene markers ANP and BNP (Chen et al., 2008; Yang et al., 2013b). These data suggest that β-AR and LPA_3_ mediate distinct effectors and, in support of this, the present study provides experimental evidence for LPA-LPA_3_ signaling being distinct from ISO-β-AR-mediated myocardial hypertrophy. It is worth noting that another important GPCR in the cardiovascular system, AT1aR, interacts with the β-AR system, since AT1aR^−/−^ mice showed remarkable repression of cardiac hypertrophy and oxidative stress in response to ISO stimulation (Zhang et al., 2007), in contrast to LPA_3_ signaling. Whether LPA_3_ contributes to myocardial hypertrophy via the related RAAS or represents a new system, awaits further study.

We found that LPA_3_ deficiency attenuated cardiac hypertrophy but aggravated cardiac dysfunction after MI. Cardiac hypertrophy involved in myocardial remodeling includes compensated hypertrophy and decompensated hypertrophy, which are associated with different signaling pathways (Tham et al., 2015). Decompensated hypertrophy results in pathological hypertrophy and heart failure, which involves the activation of NFAT signaling, β-AR-CaMKII signaling, cGMP-PKG signaling, and PKC-MAPKs signaling (Bernardo et al., 2010; Shimizu and Minamino, 2016). Overactivation of β-AR correlates with cardiotoxic outcomes such as hypertrophy and heart failure (Osadchii, 2007; Pleger et al., 2007; El-Armouche and Eschenhagen, 2009). While blockade of β-AR remarkably reduces cardiac hypertrophy and promotes heart function in mouse model (Ni et al., 2011). As Gs-cAMP-PKA and cAMP-CaMKII-Epac-NFAT are central mechanisms of β-AR-induced hypertrophy, gain- and loss- of function studies show that they are all involved in pathological hypertrophy. For example, transgenic over-expression studies indicate that Gsα (Gaudin et al., 1995), PKA (Antos et al., 2001), or NFAT(Wilkins et al., 2004) are all sufficient for inducing pathological hypertrophy and heart failure *in vivo*. Gain of CaMKII function leads to cardiac hypertrophy, while inhibition of CaMKII ameliorates myocardial hypertrophy and improves heart function (Kirchhefer et al., 1999; Ai et al., 2005; Bossuyt et al., 2008). Although, the boundary between compensated and decompensated hypertrophy is not clearly elucidated, some signaling pathways are considered to be beneficial (Tham et al., 2015; Shimizu and Minamino, 2016). For example, the insulin-like growth factor 1 (IGF1) -phosphoinositide-3-kinase (p110α)—protein kinase B (Akt) signaling pathway appears to be involved in mediating physiological heart growth such as postnatal and exercise-induced hypertrophy, but not pathological hypertrophy like that associated with pressure overload (e.g., by constricting the ascending aorta) (McMullen et al., 2003, 2004; Luo et al., 2005; Debosch et al., 2006; Kim et al., 2008). Among the mitogen-activated protein kinases (MAPKs) superfamily, ERK1/2 has been reported to mediate both adaptive (Gαq mediated) (Bueno et al., 2000) and maladaptive (via Gβγ) (Lorenz et al., 2009) processes through different phosphorylation sites within the heart. As the underlying mechanisms of LPA-induced cardiomyocyte hypertrophy involve activation of Akt and ERK-NFκB (Chen et al., 2008; Yang et al., 2013b), with Gαq-dependent signaling by LPA_3_ reported in other cell systems like vascular smooth muscle (Kim et al., 2006), we speculate that in contrast with detrimental effects of ISO-β-AR activation, LPA-LPA_3_ signaling might represent another upstream stimulus for adaptive hypertrophy.

It is well-known that GPCRs are much important drug targets. β-blockers and AngII receptor inhibitors have become widely used and effective cardiovascular medicines (Liao et al., 2004; Ouwerkerk et al., 2017). In the present study, we found that LPA_3_ deficiency led to reduced cardiac function and increased infarct size after MI. LPA_3_ also belongs to GPCR family, and our study hits that functionally enhances LPA_3_ might be a new strategy to protect heart from ischemia injury. The role of LPA signaling in cardiovascular diseases is broad. On one hand, LPA is reported to promote the progression of atherosclerotic vascular diseases (Siess et al., 1999; Kritikou et al., 2016). On the other hand, LPA has protective roles in cell survival. For example, LPA protects CD34^+^ cells from ischemia-induced apoptosis and the delivery of LPA-treated CD34^+^ cells into the infarcted heart improved cardiac function (Kostic et al., 2015). We also reported that LPA may protect cardiomyocytes from hypoxia/reperfusion-induced injury by activating LPA_3_ and suppressing mitochondrial apoptotic pathways *in vitro* (Yang et al., 2013a) and *ex vivo* (Chen et al., 2017). Here we found aggravated cardiac dysfunction causing by LPA_3_ deficiency appears to correlate with decreased hypertrophy, but there may be other, concomitant mechanisms, such as resistance to apoptosis. However, it needs further study to prove that anti-apoptosis action of LPA is responsible for myocardial protection of LPA-LPA_3_ during myocardial remodeling post-MI.

In conclusion, this study suggests that LPA_3_ participates in hypertrophy associated with myocardial remodeling post-MI, but does not appear to be involved in the induction of hypertrophy in response to β-AR stimulation *in vitro* and *in vivo*. LPA_3_ deficiency leads to dysfunction of the myocardium post-MI. Taken together, these results indicate that LPA-LPA_3_ lipid may be a novel regulator of cardiac hypertrophy, which in turn suggests that strategies aimed at LPA_3_ modulation could protect the myocardium against ischemic injury.

## Author contributions

XC, XfC, FW, and LC conceived the studies. LC, GF, TL, and SL performed the experiments. LC and FW analyzed and interpreted data. JC provided the ${\text{LPA}}_{3}^{-/-}$ mice. LC wrote, and XC, FW, JC, and GF revised the manuscript. All authors have read and approved the final manuscript.

### Conflict of interest statement

The authors declare that the research was conducted in the absence of any commercial or financial relationships that could be construed as a potential conflict of interest.
